# Soft-tissue simulation of the breast for intraoperative navigation and fusion of preoperative planning

**DOI:** 10.3389/fbioe.2022.976328

**Published:** 2022-09-28

**Authors:** Patricia Alcañiz, César Vivo de Catarina, Alessandro Gutiérrez, Jesús Pérez, Carlos Illana, Beatriz Pinar, Miguel A. Otaduy

**Affiliations:** ^1^ Computer science department, Universidad Rey Juan Carlos, Madrid, Spain; ^2^ GMV Innovating Solutions, Madrid, Spain; ^3^ Computer science department, Universidad Las Palmas de Gran Canaria, Las Palmas de Gran Canaria, Spain; ^4^ Fundación Para La Investigación Biomédica Del Hospital Universitario La Paz, Madrid, Spain; ^5^ Medical Physics department, Hospital Universitario Doctor Negrín, Las Palmas de Gran Canaria, Spain

**Keywords:** soft-tissue simulation, finite-element model, IORT, breast tumor resection, preoperative planning, navigation

## Abstract

Computational preoperative planning offers the opportunity to reduce surgery time and patient risk. However, on soft tissues such as the breast, deviations between the preoperative and intraoperative settings largely limit the applicability of preoperative planning. In this work, we propose a high-performance accurate simulation model of the breast, to fuse preoperative information with the intraoperative deformation setting. Our simulation method encompasses three major elements: high-quality finite-element modeling (FEM), efficient handling of anatomical couplings for high-performance computation, and personalized parameter estimation from surface scans. We show the applicability of our method on two problems: 1) transforming high-quality preoperative scans to the intraoperative setting for fusion of preoperative planning data, and 2) real-time tracking of breast tumors for navigation during intraoperative radiotherapy. We have validated our methodology on a test cohort of nine patients who underwent tumor resection surgery and intraoperative radiotherapy, and we have quantitatively compared simulation results to intraoperative scans. The accuracy of our simulation results suggest clinical viability of the proposed methodology.

## 1 Introduction

Computational preoperative planning of surgical interventions entails multiple benefits, in particular reduced risk to the patient and shorter interds, computational preoperative planning has seen success in applications such as radiotherapy Calvo et al. (2014), neurosurgery Prada et al. (2014); Kahl et al. (2021); Nemoto et al. (2002), or orthognathic surgery Alcañiz et al. (2021).

Image-guided surgery on the breast, on the other hand, hardly benefits from computational preoperative planning. Due to the soft-tissue nature of the breast, there is often a large deviation between the preoperative and intraoperative deformation conditions, which complicates mapping any preoperative data to the intraoperative setting. To date, clinicians resort to three suboptimal options: 1) They rely on preoperative images, which unfortunately do not represent the deformed setting during surgery; 2) they may try to predict the deformation during surgery as part of the preoperative planning, but this is hardly feasible due to the unknown details of the surgery; 3) they can capture high-quality images during surgery, but this process is slow and it requires a readily available imaging system in the operating room.

We pay particular attention to tumor resection due to breast cancer and subsequent intraoperative radiation therapy (IORT) Vaidya et al. (2005); Vaidya (2007). IORT delivers a large dose of ionizing radiation to the tumor bed during surgery, thus maximizing the dose applied to the tumor while minimizing the dose applied to nearby healthy tissue. Preoperative planning of IORT involves calculation of a cumulative dose on a volume image that faithfully represents the patient’s anatomy, and is successfully used in, e.g., neurosurgery Prada et al. (2014); Kahl et al. (2021); Nemoto et al. (2002). However, to date IORT is hardly possible on breast cancer surgery, due to the strong mismatch between preoperative and intraoperative settings. This mismatch is due to two major reasons, which cannot be predicted in advance. One major reason is surgical manipulation, such as tumor resection and retraction of mobile structures in the path of the beam. The other major reason is the insertion and motion of the IORT device (applicator).

In our work, we propose a high-performance yet accurate simulation model of the breast, to fuse preoperative information, e.g., high-quality CT images, with the intraoperative deformation setting. Our simulation method encompasses four major elements:

1) An efficient patient-specific finite-element (FEM) simulation model. A key feature of our method is to approach two major tasks of soft-tissue modeling, namely mesh preparation and mathematical modeling, in a cross-informed manner. In this way, we obtain a runtime simulation model that is accurate but efficient, as it avoids complex coupling of different anatomical elements and the need for high-resolution simulation meshes.
2) Estimation of personalized simulation parameters from multiple preoperative images. We obtain two preoperative images per patient, a CT scan and a surface scan, in two different configurations that span beyond the deformation from the preoperative to the intraoperative setting. By optimizing mechanical tissue parameters that accurately fit this deformation, we obtain a personalized soft-tissue model that is able to predict intraoperative deformations.
3) Deformation of a high-quality breast model from a preoperative CT scan to the intraoperative configuration. With the high-accuracy simulation model estimated as described above, we deform the preoperative breast model to the intraoperative configuration. Once in this intraoperative configuration, it is possible to execute IORT dose planning.
4) Real-time simulation of breast deformation, for high-accuracy intraoperative navigation. We further optimize the mesh complexity of the breast simulation model to enable real-time deformation during intraoperative navigation. The real-time simulation can take as input tracker data of the IORT applicator, and use these as boundary conditions to compute the breast deformation.

In Section 2.1 we list the input data of both our simulation methodology and experimental validation. In Section 2.2 we describe the FEM simulation model and how it is configured from patient-specific data. Then in Section 2.3 we describe the estimation of soft-tissue parameters.

In Section 3.2 we demonstrate the accuracy of breast deformation from preoperative to intraoperative settings. We have tested our methodology on a cohort of nine patients who underwent tumor resection surgery and IORT. For all these patients, we obtained preoperative data (a CT-scan in supine position and a surface scan in sitting position) as well as intraoperative data (a surface scan in surgical position and the position of the IORT applicator). We have estimated a personalized simulation model for each patient, and we have evaluated the accuracy of the deformation to the intraoperative configuration.

Finally, in Section 3.3, we demonstrate real-time simulation of breast deformation due to motion of the IORT applicator, suitable for intraoperative navigation.

Before describing the methods and results of our work, next we discuss related work. Much of the previous work on planning for breast interventions and IORT has focused on bridging the gap between preoperative data and the operative setting. We discuss work that follows different approaches, either navigation techniques, simulation techniques, or fusing preoperative images (CT, MRI, or mammography) with the intraoperative setting.

### 1.1 Related work-navigation

Navigation is a problem that has received important commercial attention. There are multiple available commercial navigation systems, but they typically target specific applications. Some of the most prominent examples are: SurgicEye, for imaging of sentinel lymph; Aesculap, for orthopaedics; or Medtronic, for neurosurgery. Unfortunately, none of these systems combines preoperative imaging, such as CT or MRI, with intraoperative navigation. Therefore, they cannot accommodate preoperative planning data.

In addition to commercial systems, there is also important research on navigation. For the particular problem of breast cancer, there are works that have proposed navigation solutions for breast biopsy Treepong et al. (2014), and for sonography-guided breast surgery Ungi et al. (2016). In this project, we propose matching the input anatomy to intraoperative tracking data and use soft-tissue simulation methods to estimate the deformation. Based on this deformation, one could compute the required dose distribution solution on a deformed high-resolution image of the patient’s anatomy, overcoming the problem of geometric mismatch between the preoperative images and the operative setting during irradiation.

### 1.2 Related work-soft-tissue models

The first task in devising a soft-tissue model of the breast is to segment and mesh its geometry. As we discuss later in Section 2.2, we follow the most common approach of obtaining a CT-scan in supine position, and segmenting and meshing this CT-scan. However, the scanning position differs from the surgical position, and we must deform the model. Recently, citeMazier2021 have introduced a different approach, as they initialize the geometry of the breast directly in surgical position. They do this by quickly morphing a geometric breast template to a surface scan obtained in surgical position. As their template is endowed with surgical drawings that aid in planning, they also obtain personalized surgical drawings. In a way, we solve the same morphing task, but our model also enjoys mechanical response, while theirs is only geometric. Therefore, we can further deform the breast model, e.g., for runtime navigation.

Multiple previous works have attempted high-quality FEM simulation of the soft-tissue in the breast. Those works can be largely classified into three groups based on the specific problems that they target: compression of the breast under two rigid plates Tanner et al. (2006b); Azar et al. (2001); Pathmanathan et al. (2008); Han et al. (2012), deformation due to gravity loading Han et al. (2011); Tanner et al. (2006b); Danch-Wierzchowska et al. (2016) and surgical planning or outcome prediction Lapuebla-Ferri et al. (2011); del Palomar et al. (2008).

The majority of the methods execute FEM analysis using tetrahedral meshes; however, hexahedral meshes have also been used Pathmanathan et al. (2008); Tanner et al. (2006b). In recent works, hexahedral meshes are gaining popularity, at least on the brain and other organs Miller et al. (2010); Wittek et al. (2010); Alkhatib et al. (2022), specially as they are complemented with methods that support complex geometries Bui et al. (2019). The various methods also differ in terms of the discretization complexity of the models, ranging from hundreds of elements Tanner et al. (2006b), to thousands of elements Pathmanathan et al. (2008), or even tens of thousands of elements Tanner et al. (2006a); Han et al. (2012). We tune the mesh complexity to optimize the balance between accuracy and performance depending on the target problem.

### 1.3 Related work-material properties

In the simulation of soft tissue in the breast, the various previous works differ in their choice of material model as well. Some authors used linear elastic materials (e.g., Tanner et al. (2006a)), while others used nonlinear hyperelastic materials (e.g., Tanner et al. (2006b)). Comparative studies indicate that hyper-elastic materials provide higher accuracy, which is expected given the potential large deformation of the breast. In particular, the incompressible Neo-Hookean material provided the best results when comparing deformations in standing and prone positions Griesenauer et al. (2014). Some works have analyzed the parameterization of the incompressible Neo-Hookean material, in particular the stiffness for the energy term of the first invariant (typically denoted as *C*_1_ or *μ*/2, with the shear modulus *μ* computed as *E*/2 (1 + *ν*) in terms of Young modulus *E* and Poisson’s ratio *ν*). Optimal values range between *C*_1_ = 0.13 kPa Tanner et al. (2006a) and *C*_1_ = 0.08 kPa Rajagopal et al. (2008). Griesenauer et al. Griesenauer et al. (2014) recommend using the average value of *C*_1_ = 0.105 kPa.

One important conclusion from previous work is the large variability in material parameters across subjects. Using deterministic approaches for parameter adjustment has proven significantly limited, and sensitivity analysis and uncertainty quantification are necessary to obtain results including confidence intervals Biehler et al. (2015); Sankaran and Marsden (2011); Hauseux et al. (2017, 2018); Rego et al. (2021). Motivated by this variability, we carry out a personalized fitting of the soft-tissue properties of the breast.

Beyond the material model, previous works also differ in terms of the degree of heterogeneity in the breast models. Some works Han et al. (2012); Tanner et al. (2006b,a) have used different tissue classes: fibro-glandular, fat and skin. Other works Danch-Wierzchowska et al. (2016), on the other hand, differentiate only between internal (muscle, thorax and internal organs) and external tissue (breast tissue including fat and surrounding skin).

### 1.4 Related work-adaptive meshes

For one of our target applications, runtime navigation, we use adaptive simulation meshes that maximize mesh resolution at areas of interest. Adaptive meshing is a long-standing idea, even in the context of dynamic mesh adaptivity Wu et al. (2001). We use a fixed adaptive mesh, as the region of interest, i.e., the region surrounding the tumor, is known in advance. Recent works evaluate the actual error introduced by discretization from a rigorous mathematical perspective Wittek et al. (2010); Bui et al. (2018a, 2019, 2018b); Duprez et al. (2020). Interestingly, the actual resolution and distribution of the discretization may vary considerable depending on the error metric used. Therefore, our meshing approach could be further enhanced by introducing a goal-oriented error estimate.

## 2 Materials and methods

In this section, we describe how we create patient-specific soft-tissue models of the breast. In our modeling methodology we follow two fundamental principles:

• We approach mesh preparation and mathematical modeling in a cross-informed manner, to produce simulation models that maximize efficiency.
• We obtain patient-specific data in multiple configurations, to estimate best-matching soft-tissue parameters.

The section starts with a description of the input data. Next, it describes the mathematical simulation model, the initialization of the model from imaging data, and the definition of boundary conditions. Finally, the section covers the estimation of soft-tissue parameters.

### 2.1 Input data

To create a personalized model of the patient’s breast, we leverage multiple input data. First, we acquire a high-quality CT scan in a pose similar to the surgical configuration. In particular, we use a CT scan in supine position with arms lifted. Using this CT scan, we extract the 3D simulation model, as we discuss in more detail in the next section.

However, using the CT scan alone poses several challenges. While the configuration is close to the surgical position, it is not fully accurate, as the surgical bed may slightly reoriented. But most importantly, the CT scan does not capture the mechanical response of the breast tissue.

To estimate soft-tissue mechanical parameters, we capture two 3D surface scans of the patient’s torso: one 3D scan in the same supine position as the CT scan, $S_{\text{supine}}$ , and another 3D scan in a relaxed sitting position, $S_{\text{sitting}}$ . The deformation between these two 3D scans is larger than the deformation during surgery, and it allows us to robustly estimate simulation parameters, as we discuss later in Section 2.3. We use 3D scans instead of CT scans to minimize radiation on the patient.

**FIGURE 1:**
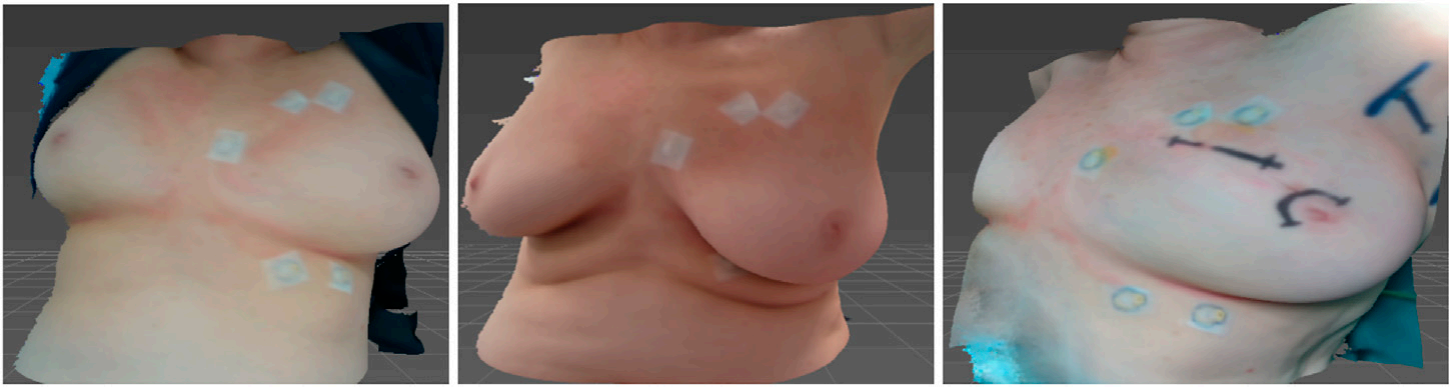
3D surface scans for the same patient in different positions. From left to right: supine position with arms lifted, which corresponds to the regular CT scanning position; sitting position, which we use for parameter estimation; surgical position, used for validation.

Furthermore, for validation purposes, we also acquire a 3D scan during surgery. Note that this last scan is not part of the modeling and simulation methodology; it was captured only for validation as part of this study. Figure 1 shows all three 3D scans captured for each patient.

### 2.2 Simulation model

**FIGURE 2:**
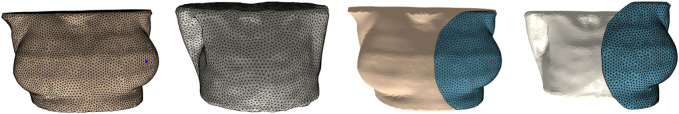
Main components of the simulation model. From left to right: (i) segmentation and meshing of surface skin from the CT-scan, (ii) segmentation and meshing of the chest wall from the CT-scan, (iii) simulation mesh, obtained by selecting a portion of the skin corresponding to the breast, (iv) combination of the simulation mesh and the chest wall that acts as boundary condition.

Our approach to model the breast is to identify soft and rigid anatomical elements in the region of interest, as shown in Figure 2, define efficient coupling boundary conditions between them, and solve the tissue deformation using FE analysis. This approach mimics previous work for the simulation of orthognathic surgery Alcañiz et al. (2021), but applied to breast simulation. We follow this approach to simulate both the interaction between the soft-tissue and stiff or bone tissue, as well as to handle contact between the breast soft-tissue and the IORT applicator.

We start by segmenting rigid or stiff tissue in the breast area in the input CT scan. In particular, we segment jointly the ribs, intercostal muscle and the pectoralis muscle. These all form a single continuous rigid body in the simulation, which we refer to as chest wall in the rest of the text. In addition, we segment separately the remaining skin tissue. Finally, we limit the simulation mesh to a region of interest centered on the breast, by picking a central point and a radius. We use 3D Slicer for segmentation tasks and to create the initial mesh. However, this mesh is too detailed, and then we use Meshmixer to obtain the final simulation mesh.

To build the FE simulation model, we integrate a strain energy density and a gravity potential on the volume of the breast soft-tissue. Instead of rotating the chest wall to represent different configurations of the torso, we keep the chest wall fixed and we rotate the gravity vector. Furthermore, this approach allows us to circumvent the estimation of an undeformed configuration for the breast soft-tissue. The breast is deformed in supine position where the mesh is initialized, due to the action of gravity forces, but we define the undeformed position simply by applying a negative gravity vector. Simulating an arbitrary rotation of the torso amounts to first applying this negative gravity vector and then adding the rotated gravity.

We compute the gravity potential as

$$V_{\text{grav}}=-\int_{\Omega }\rho \;g^{T}\;x\;d\Omega ,$$ (1)

with *ρ* the breast mass density (which we define as the density of water), *g* the gravity vector for the configuration to be simulated, and *x* the deformationdisplacement of the tissue point.

We define the elastic energy as

$$V_{\text{soft}}=\int_{\Omega }\Psi \left(F\right)\;d\Omega ,$$ (2)

with Ψ(*F*) the constitutive model of the breast soft-tissue and *F* the deformation gradient. Following previous work, we use a Neohookean model $\Psi \left(F\right)=\frac{\mu }{2}\;\left(\text{trace}\left(F^{T}\;F\right)-3\right)-\mu \;\log\left(\det\left(F\right)\right)+\frac{\lambda }{2}\;{\left(\log\left(\det\left(F\right)\right)\right)}^{2}$ . Recall that *C*_1_ = *μ*/2, which is the material parameter typically estimated in the literature.

The simulation mesh defines a discretization of the deformationdisplacement field *x* on the breast mesh domain Ω, and we approximate the integrals of elastic energy and gravity potential using finite elements. In our work, we have used tetrahedral finite elements. We also separate degrees of freedom coupled to the rigid parts, **x**_rigid_, and free degrees of freedom, **x**_free_. Then, the computation of static breast deformationsdisplacements **x** can be posed as an optimization problem, with the rigid parts as boundary conditions:

$$x=\arg\underset{x_{\text{free}}}{\min}V_{\text{soft}}\left(x_{\text{free}},x_{\text{rigid}}\right)+V_{\text{grav}}\left(x_{\text{free}},x_{\text{rigid}},g\right).$$ (3)

We solve this optimization to predict the breast deformation every time we change the boundary conditions **x**_rigid_. We do this using Newton’s method, with a conjugate gradient solver for the solution of linear systems of equations. We refer the reader to Alcañiz et al. (2021) for details.

### 2.3 Estimation of soft-tissue parameters

For preoperative planning and/or intraoperative navigation, the breast soft-tissue must be simulated in configurations defined by the intraoperative transformation of the clinical bed. While this configuration is similar to the supine position of the CT-scan, as shown in Figure 1, we estimate soft-tissue parameters by fitting a larger deformation. This approach ensures that the deformations in the intraoperative setting do not extrapolate to far unknown situations. In particular, we estimate soft-tissue parameters to best match the transformation of the breast from supine to sitting position, as shown in Figure 1.

We choose to estimate a homogeneous breast soft-tissue material, defined by its Young modulus, to avoid overfitting problems. As we discuss later in the results section, this parameterization provides sufficient accuracy for our target applications.

**FIGURE 3:**
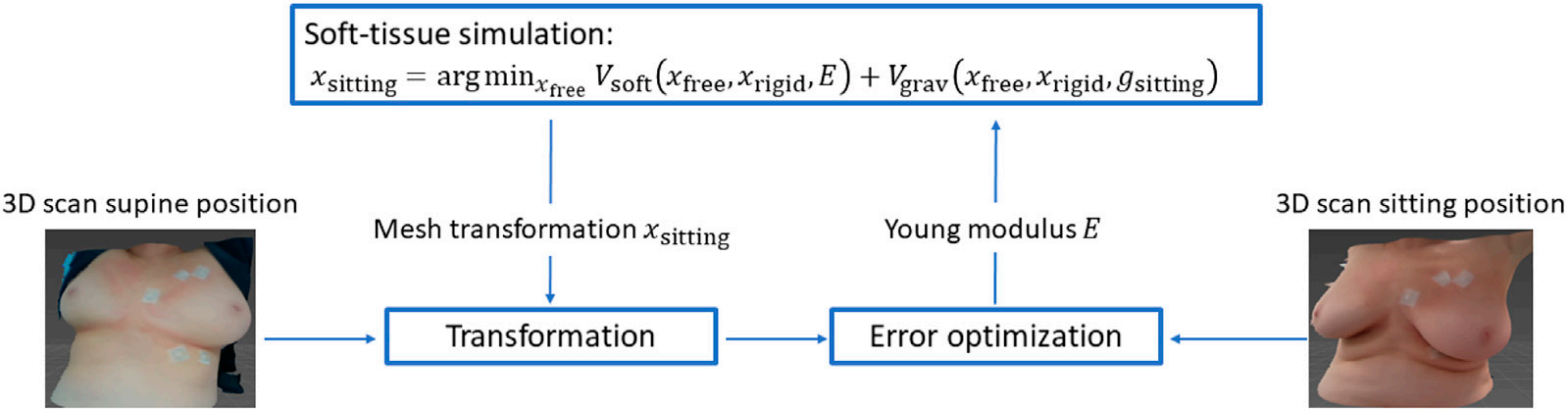
We estimate soft-tissue parameters of the breast model (i.e., its Young modulus) by solving an optimization problem. We take the boundary conditions of the sitting configuration and we compute the deformation of the breast from supine to sitting configuration. Using this deformation, we transform the supine-position 3D scan, we register it to the sitting-position 3D scan, and we use the registration error to further optimize the material parameters.

We initialize the Young modulus to a value of *E* = 0.609 kPa, corresponding to the average value of *C*_1_ = 0.105 kPa from the literature (as discussed in Section 1.3), and Poisson’s ratio of *ν* = 0.45. Then we execute a patient-specific optimization process, to estimate the Young modulus such that a tranformation of the 3D scan in supine position best matches the 3D scan in sitting position. We depict this optimization process schematically in Figure 3. Formally, the optimization is expressed as follows:

$$\begin{array}{l} E=\arg\underset{E}{\min}|T\left(S_{\text{supine}},x_{\text{sitting}}\left(E\right)\right)-S_{\text{sitting}}{|}^{2},\\ x_{\text{sitting}}=\arg\underset{x_{\text{free}}}{\min}V_{\text{soft}}\left(x_{\text{free}},x_{\text{rigid}},E\right)+V_{\text{grav}}\left(x_{\text{free}},x_{\text{rigid}},g_{\text{sitting}}\right). \end{array}$$ (4)

Given a tentative Young modulus *E* and the gravity vector in sitting position *g*_sitting_, we compute the deformationdisplacement of the breast *x*_sitting_ by solving the simulation problem (Eq. 3). Using this deformation, we transform (denoted by function *T*) the 3D scan in supine position $S_{\text{supine}}$ , and we register the result to the 3D scan in sitting position $S_{\text{sitting}}$ , to compute a registration error. Based on this registration error, we further optimize the Young modulus *E*.

Our modeling and estimation approach is limited by its treatment of gravity, described above in Section 2.2. In supine position the breast is deformed, under internal stress. Due to the nonlinearity of the tissue, this underlying stress produces an error as the gravity vector is rotated. A more sound approach would require estimating the rest position of the breast together with the soft-tissue parameters, such that both the sitting and supine deformations are met under the appropriate boundary conditions. Other works have already explored undoing the effect of gravity for breast modeling Mazier et al. (2022); Mîra et al. (2018). However, while potentially more accurate, this approach introduces a high number of degrees of freedom in the optimization and makes it substantially more nonlinear. Since the supine position is close to the surgical position, we find approach to be a sufficient compromise. This is validated by our experiments.

## 3 Experiments and results

In this section, we discuss the experiments carried out to validate our simulation methodology. We start with a detailed description of the test cohort and the estimation of soft-tissue parameters following the approach described in Section 2.3. Then, we perform quantitative evaluation of the accuracy and performance of the simulation model, on the two target deformation problems: deformation of the breast from preoperative configuration to intraoperative configuration, and real-time deformation for intraoperative navigation. Deformation to the preoperative configuration enables cross-validation of the soft-tissue model, as we measure its accuracy on a test configuration not used for model training.

### 3.1 Test cohort and parameter estimation

We performed the study on a cohort of nine patients; all of them underwent tumor resection surgery and IORT, and we acquired preoperative and intraoperative scans (one CT-scan and three 3D surface scans, as discussed previously in Section 2.1). All data was acquired at Hospital Universitario Doctor Negrín, with approval of the hospital’s ethics committee, and with informed consent from all the patients.

**FIGURE 4:**
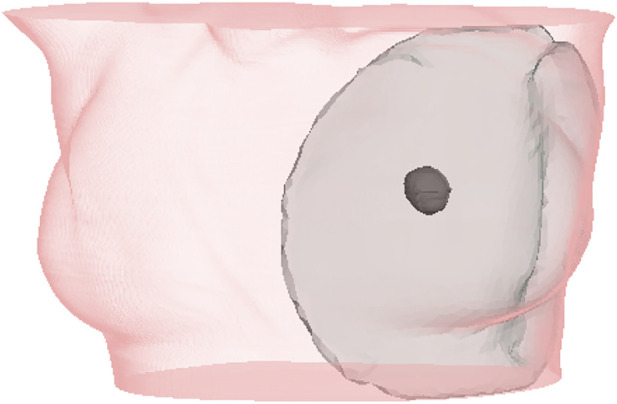
Example case for patient #2 in the cohort. The image highlights the simulated area and the location of the tumor within the breast.

Table 1 lists details of all patients in the cohort. The age range was 51–84 years, tumors were located on both left 6) and right breasts (3), in different locations on the breast (some centered, some near skin and/or the chest wall), and the size of breasts ranged from small to large. Figure 4 highlights the simulation mesh and the location of the tumor for one particular patient in the cohort (patient #2). Table 1 also lists the inclination of the surgical in the intraoperative setting. This information was used for defining the gravity vector *g* in the deformation from the preoperative to the intraoperative configuration.

**TABLE 1:** Characteristics of the nine patients analyzed in the study, including age, breast characteristics, tumor position, anteroposterior (AP) and lateral (LAT) inclination of the surgical bed, mesh size (in tetrahedra) of the simulated volume, and estimated Young modulus.

ID	Age	Breast	Size	Tumor Position	AP (°)	LAT (°)	Mesh (tets)	Young (kPa)
1	60	Left	Medium	Near chest wall	5	4	8,227	0.8
2	55	Left	Large	Near skin	5	4	11,672	0.9
3	83	Right	Medium	Centered	5	4	11,828	1.2
4	56	Left	Medium	Centered	7	7	12,235	0.6
5	53	Left	Medium	Centered	7	4	11,739	0.5
6	51	Left	Small	Near skin and chest wall	9	5	4,997	1.2
7	84	Left	Small	Near skin and chest wall	6	7	6,712	0.4
8	69	Right	Small	Near skin and chest wall	9	5	5,444	0.3
9	61	Right	Large	Centered	10	6	12,947	2.0

Following the procedure described in Section 2.3, and using the preoperative 3D surface scans in supine and sitting positions, we estimate the Young modulus for the breast simulation models of all patients. Table 1 includes the size of the simulation mesh for each patient, as well as the Young modulus resulting from the estimation.

### 3.2 Deformation to the intraoperative setting and cross-validation

One of the applications of the soft-tissue breast simulation is to deform a preoperative CT scan to the intraoperative configuration, and thus plan the dose for IORT Valdivieso-Casique et al. (2015). Here, we measure the error produced by our model in this deformation. As the intraoperative data was not used for estimating the soft-tissue model, this test serves for cross-validation.

**FIGURE 5:**
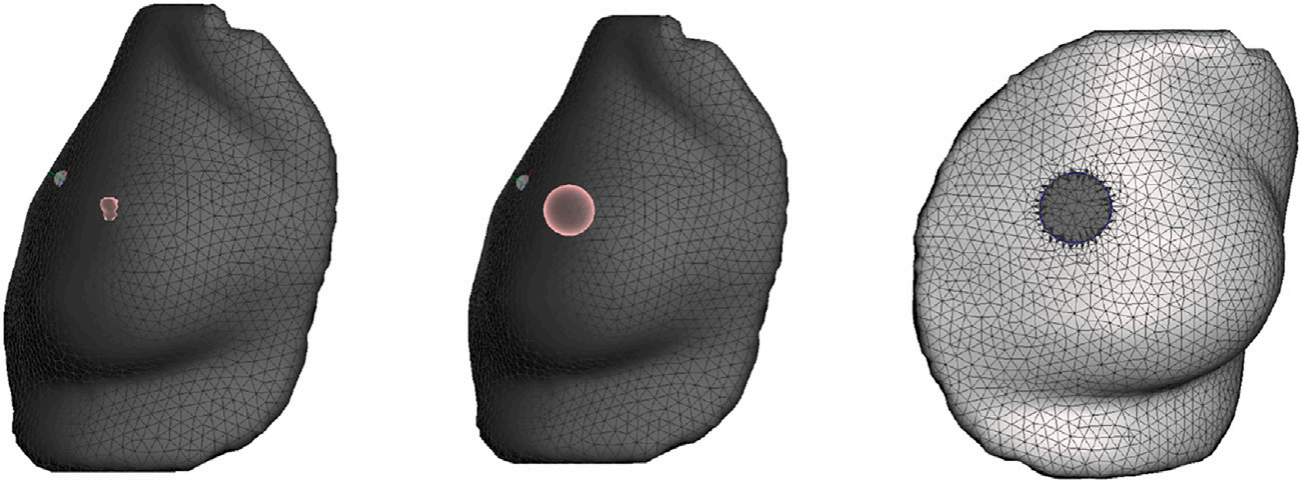
From left to right: (i) Location of tumor highlighted on top of the chest wall; (ii) 2-cm-radius sphere centered on the tumor; (iii) region of interest on the skin surface produced by intersecting the sphere.

The deformation error is most relevant in the volume near the tumor, as this is the location where the radiotherapy dose will be planned. However, we lack a CT scan in intraoperative configuration, hence we cannot locate the tumor in this configuration. As an alternative, we use the preoperative and intraoperative surface 3D scans to measure deformation error. Specifically, we locate the tumor on the preoperative CT scan, we find the point on the surface that is closest to the tumor, and then we identify a region of interest (ROI) of roughly 2-cm-radius centered on this point, as shown in Figure 5. We chose this ROI following advice from clinical experts, as a sufficiently large region in the proximity of the tumor.

Table 2 shows the deformation error from preoperative to intraoperative configuration on the ROI, for all patients in the cohort. In all cases, the error within the ROI is below 5 mm. We initialize the simulation mesh in preoperative supine position, we apply the orientation corresponding to the intraoperative configuration to each patient (see Table 1), and we compute the deformation of the breast tissue. With this deformation, we transform the preoperative 3D surface scan, we register the result to the intraoperative 3D surface scan, and we measure the signed-distance error within the ROI. Table 2 shows the input simulation mesh in supine position, as well as the deformed mesh in the intraoperative configuration. The fitting error could be reduced by allowing heterogeneous soft-tissue parameters across the breast, but this could easily incur in overfitting error, due to the scarcity of deformation examples.

**TABLE 2:** Quantitative evaluation of the deformation error between preoperative and intraoperative setting. We initialize the simulation mesh in preoperative supine position, we apply the orientation corresponding to the intraoperative configuration to each patient (see Table 1), and we compute the deformation of the breast tissue. With this deformation, we transform the preoperative 3D surface scan, we register the result to the intraoperative 3D surface scan, and we measure the error on the region-of-interest (ROI) closest to the tumor. The colors indicate signed-distance error (in mm) between the simulation result and the actual intraoperative 3D scan. In all cases, the error in the ROI is below 5 mm.

Tumor location	Error	Tumor location	Error	
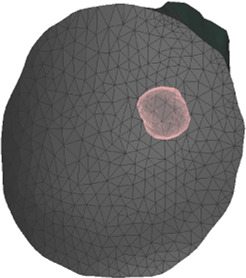	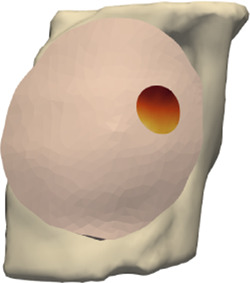	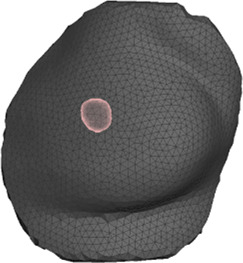	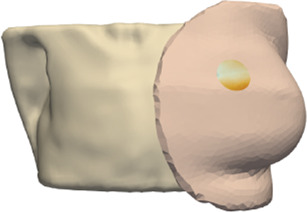	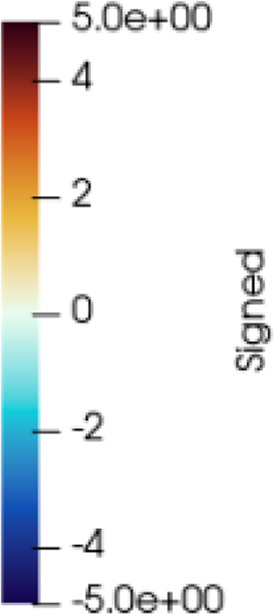
Patient 1		Patient 2		
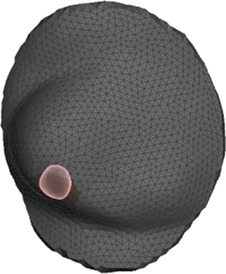	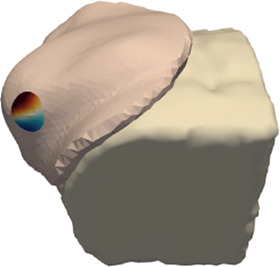	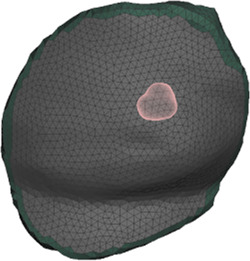	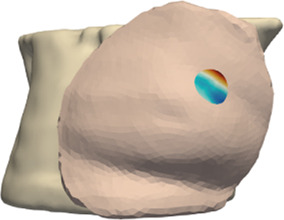	
Patient 3		Patient 4		
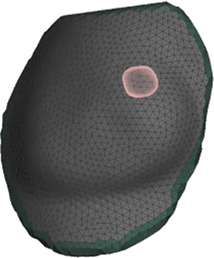	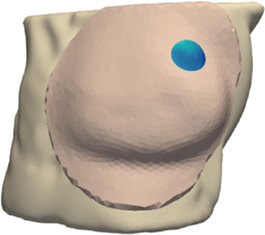	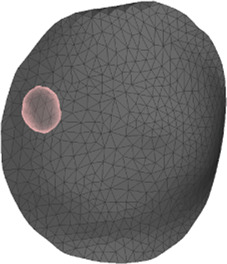	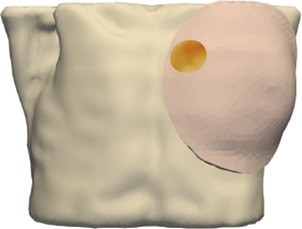	
Patient 5		Patient 6	
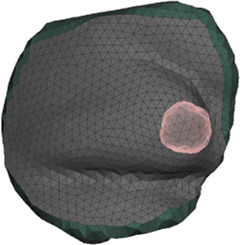	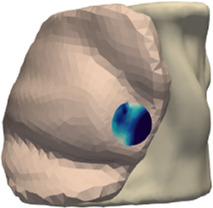	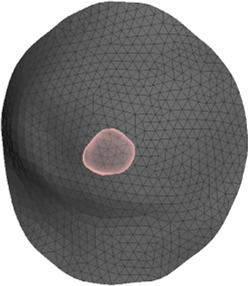	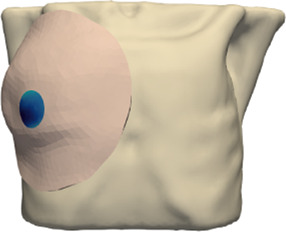
Patient 7		Patient 8	
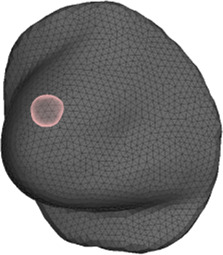	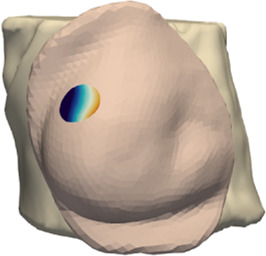		
Patient 9			

### 3.3 Real-time deformation for intraoperative navigation

**FIGURE 6:**
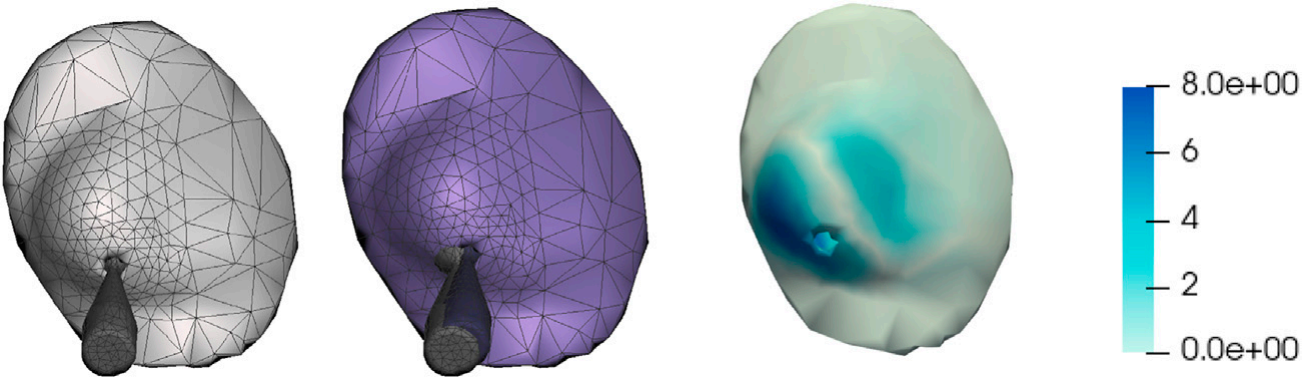
Quantitative evaluation of real-time deformation for intraoperative tracking. From left to right: (i) Initial simulation mesh (in grey), showing the IORT applicator; (ii) Simulation mesh (in purple) after moving the applicator a total distance of 8.7 mm, with the initial and final applicators overlaid; (iii) Per-vertex distances between initial and final position (in mm). The simulation is executed at a speed that allows a motion of the applicator of 29 mm/s.

The other major application of the soft-tissue breast simulation is to enable real-time tracking of the tumor during navigation for IORT. We start with the tumor in the preoperative configuration, and then we transform it to the intraoperative configuration based on the orientation of the surgical bed as discussed in the previous section. During IORT, it is possible to track in real-time the position and orientation of the IORT applicator, and we use this transformation as boundary conditions for real-time simulation of breast deformation, and hence track the tumor’s location. Figure 6 depicts an example IORT applicator mesh inserted in the breast simulation mesh.

To deform the breast from preoperative to intraoperative setting, we use a high-resolution simulation mesh, as this deformation is not necessarily interactive. However, for real-time tracking, deformations must be interactive. Therefore, we have validated the ability to compute real-time deformations with sufficient accuracy.

For this test, we have used the simulation mesh of patient #3, but we have downsized the original simulation mesh from 11,828 tetrahedra to 1,581 tetrahedra. We have selected this resolution by trial-and-error, to hit a good performance-accuracy trade-off. As discussed in Section 1.4, more advanced adaptive meshing methods could be used, e.g., considering goal-based error metrics. We have designed a motion of the applicator of roughly 8.7 mm (5 mm in each coordinate axis), subdivided into 10 steps. For each step, we have solved the deformation problem of the breast soft-tissue, as described in Section 2.2, using the configuration of the rigid applicator as boundary conditions.

Each simulation step was computed in 30 ms, on a commodity processor. This performance indicates that real-time simulation can be sustained if the velocity at which the clinician moves the applicator is under 29 mm/s, which is well above the practical needs. Figure 6 shows the initial simulation mesh of this test, including the applicator; the final mesh after moving the applicator 8.7 mm, and per-vertex distances between both meshes.

## 4 Discussion

In this work, we have described a methodology for modeling and simulation of the breast. The focus of our methodology is to find a good balance between simulation accuracy and performance, allowing fast updates of the breast soft-tissue deformation even interactively. We have confirmed that the method is applicable to a diverse range of breast sizes and tumor morphologies.

In the paper, we have performed preliminary testing toward two possible applications of fast soft-tissue simulation. Full development of these applications would require, however, additional technology components.

For preoperative planning of intra-operative radiotherapy, breast soft-tissue simulation could be used in the following way. The simulation model should be meshes in supine position, based on the available CT-scan, as we do. Then, the simulation model is deformed from supine to surgical position, as done in our experiments described in Section 3.2. Using this deformation, it is possible to deform the input CT-scan, using a volume image resampling method Gascon et al. (2013); Aguilera et al. (2015). Radiotherapy planning could be executed in two possible ways. One approach would be simulate dose planning on the pre-operative anatomy Valdivieso-Casique et al. (2015) and then transform the dose planning along with the CT-scan. Another approach would be to execute fast dose simulation on the transformed CT-scan, perhaps initialized by the transformed dose.

For intraoperative navigation, breast soft-tissue simulation could be used in the following way. A tracking method can be used to obtain in real-time the 3D configuration of surgical tools. This 3D configuration can be used as boundary conditions for soft-tissue simulation, as shown for the IORT applicator in our experiments described in Section 3.3. With fast soft-tissue simulation, the deformation of the breast is updated interactively, and the location of the tumor can be displayed to the surgeon together with the tracked configuration of the surgical tool, e.g., using augmented reality displays Azimi et al. (2012).

The various applications of runtime breast simulation are the main lines for future work. In addition to developing and integrating the complementary technology components discussed above, they require formal clinical testing and validation beyond the scope of this paper.

From a pure methodological perspective, our work also admits future improvements. One of the possible improvements, for higher accuracy of the breast simulation model, is to consider a heterogeneous tissue distribution. However, as mentioned earlier, estimating a more complex tissue model also requires more training data, i.e., breast scans under different deformations. One of the relevant aspects of our modeling methodology is that meshing and mechanical modeling are cross-informed, such that the couplings between anatomical elements can be efficiently simulated. Cross-information of these two aspects could be raised to yet another level, through adaptive meshes that are material aware. This, however, would require a combined solution to the meshing and material estimation problems. Another possible improvement is to maximize the efficiency of the quasi-static simulation method, by designing a faster solver tailored to the problem at hand. Finally, yet another possible improvement is to fully automate the creation of the soft-tissue model from the input data, thus reducing the practical overhead to deploy the solution in practice. This could be done through data-driven methods based on machine learning, which have seen recent success on soft-tissue modeling problems Mendizabal et al. (2019); Meister et al. (2020).

## Data Availability

The raw data supporting the conclusions of this article will be made available by the authors, without undue reservation.
